# Bis(*N*,*N*′-diphenyl­benzamidinium) fumarate

**DOI:** 10.1107/S1600536810017708

**Published:** 2010-06-09

**Authors:** Liana Orola, Mikelis V. Veidis

**Affiliations:** a University of Latvia, Kr. Valdemara 48, Riga, LV 1013, Latvia

## Abstract

The crystal structure of the title compound, 2C_19_H_17_N_2_^+^·C_4_H_2_O_4_^2−^, consists of centrosymmetric trimers built up of two crystallographically independent *N*,*N*′-diphenyl­benzamid­in­ium cations and one fumarate dianion, which is located on a centre of inversion. The components of the trimers are linked by N—H⋯O hydrogen bonding. In the cation, the outer rings make dihedral angles of 53.66 (5) and 78.38 (5)° with the central ring. The two outer rings make a dihdral angle of 81.49 (5)°.

## Related literature

For the structure of *N*,*N*′-diphenyl­benzamidine, see: Alcock *et al.* (1988 ▶) and for the structure of *N*,*N*′-diphenyl­benzamid­in­ium nitrate, see: Barker *et al.* (1999 ▶). For metal complexes of *N*,*N*′-diphenyl­benzamidine, see: Davies *et al.* (2001 ▶); Jiang *et al.* (2005 ▶); Cotton *et al.* (1996 ▶, 1997 ▶).
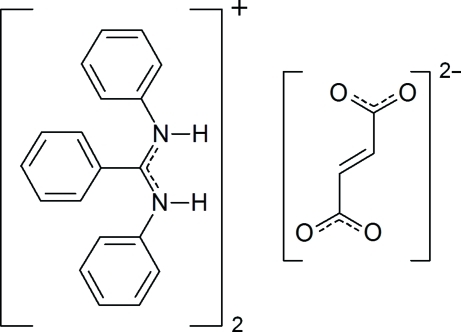


## Experimental

### 

#### Crystal data

2C_19_H_17_N_2_^+^·C_4_H_2_O_4_^2−^
*M* *_r_* = 330.39
Monoclinic, 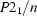

*a* = 10.5972 (3) Å
*b* = 8.8275 (3) Å
*c* = 18.7710 (7) Å
β = 102.346 (1)°
*V* = 1715.36 (10) Å^3^
*Z* = 4
Mo *K*α radiation
μ = 0.08 mm^−1^
*T* = 120 K
0.72 × 0.61 × 0.44 mm

#### Data collection

Bruker APEXII CCD area-detector diffractometer
Absorption correction: multi-scan (*APEX2*; Bruker, 2006 ▶) *T*_min_ = 0.95, *T*_max_ = 0.96
41010 measured reflections
5009 independent reflections
4976 reflections with *I* > 2σ(*I*)
*R*_int_ = 0.020

#### Refinement

*R*[*F* ^2^ > 2σ(*F* ^2^)] = 0.042
*wR*(*F* ^2^) = 0.107
*S* = 1.00
4876 reflections
226 parameters
H-atom parameters constrained
Δρ_max_ = 0.37 e Å^−3^
Δρ_min_ = −0.21 e Å^−3^

### 

Data collection: *APEX2* (Bruker, 2006 ▶); cell refinement: *SMART* (Bruker, 2006 ▶); data reduction: *SMART*; program(s) used to solve structure: *SIR92* (Altomare *et al.*, 1994 ▶); program(s) used to refine structure: *CRYSTALS* (Betteridge *et al.*, 2003 ▶); molecular graphics: *ORTEP-3 for Windows* (Farrugia, 1997 ▶); software used to prepare material for publication: *CRYSTALS*.

## Supplementary Material

Crystal structure: contains datablocks global, I. DOI: 10.1107/S1600536810017708/nc2180sup1.cif

Structure factors: contains datablocks I. DOI: 10.1107/S1600536810017708/nc2180Isup2.hkl

Additional supplementary materials:  crystallographic information; 3D view; checkCIF report

## Figures and Tables

**Table 1:** Hydrogen-bond geometry (Å, °)

*D*—H⋯*A*	*D*—H	H⋯*A*	*D*⋯*A*	*D*—H⋯*A*
N19—H4⋯O3	0.88	1.79	2.673 (1)	178
N12—H9⋯O4	0.90	1.74	2.634 (2)	172

**Fig. 1.**
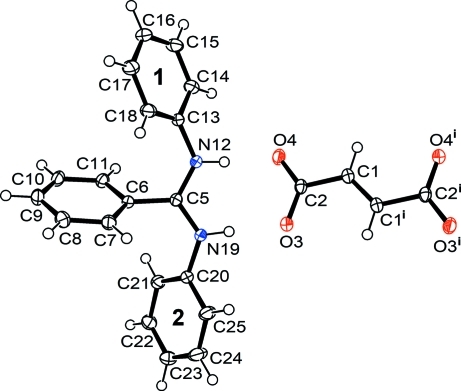
The molecular structure of the title compound with labelling and displacement ellipsoids at the 50% probability level (symmetry code for equivalent atoms: i = -x+2, -y, -z+2). 1 and 2 distinguish the two independent terminal rings.

**Fig. 2.**
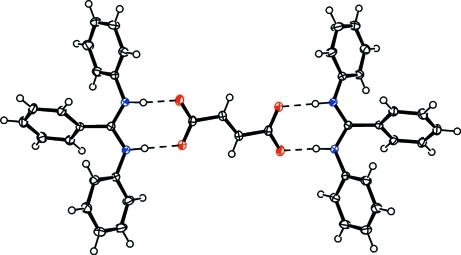
Crystal structure of the title compound with view of the trimers (hydrogen bonding is indicated by dashed lines).

## supplementary crystallographic information

### Comment

*N*,*N*'-Diphenylbenzamidine is widely used as a ligand in metal complexes and can act as a bridging (Jiang *et al.*, 2005) or a bidentate (Davies *et al.*, 2001) ligand. An important feature of many complexes with bridging bonding mode is metal-metal bond formation in dinuclear compounds (Cotton *et al.*, 1997; Cotton *et al.*, 1996). The crystal structure of *N*,*N*'-diphenylbenzamidine (Alcock *et al.*, 1988) and its nitrate salt (Barker *et al.*, 1999) have been reported previously. As part of an investigation of cocrystal synthesis, the *N*,*N*'-diphenylbenzamidine fumarate salt was obtained and the crystal and molecular structure was determined to identify the product. The asymmetric unit consist of one *N*,*N*'-diphenylbenzamidinium cation and half a fumarate dianion, which is located on a centre of inversion (Fig. 1). In the title compound there is a small difference in conformation of amidinium cation compared to *N*,*N*'-diphenylbenzamidine nitrate. In the amidinium cation the plane of the central phenyl ring is twisted by 53.7° and 78.4° with respect to the plane of the terminal phenyl rings 1 and 2 (Fig. 1). These values are slightly different from that in *N*,*N*'-diphenylbenzamidine nitrate in which a dihedral angle of 65.8° is observed (Barker *et al.*, 1999). In the crystal structure of the title compound the amidinium cations are connected by the fumarate dianions via intermolecular N–H···O hydrogen bonds into trimers that are located on centres of inversion (Fig. 2 and Table 1).

### Experimental

The title compound was prepared by dissolving *N*,*N*'-diphenylbenzamidine (0.054 g, 0.2 mmol) and fumaric acid (0.012 g, 0.1 mmol) in 5 ml of hot ethanol. Slow evaporation of the solution resulted in the formation of colorless prisms.

### Refinement

All H atoms were located in a difference map, but were positioned with idealized geometry and refined with soft restraints on the bond lengths and angles to regularise their geometry (C—H in the range of 0.93–0.98 and N—H in the range of 0.86—0.89 Å) and with U_iso_(H) in the range 1.2-1.5 times U_eq_ of the parent atom.

### Crystal data

**Table:**

2C_19_H_17_N_2_^+^·C_4_H_2_O_4_^2^^−^	*F*(000) = 696.00
*M**_r_* = 330.39	*D*_x_ = 1.279 Mg m^−^^3^
Monoclinic, *P*2_1_/*n*	Mo *K*α radiation, λ = 0.71073 Å
Hall symbol: -P 2yn	Cell parameters from 9851 reflections
*a* = 10.5972 (3) Å	θ = 3–30°
*b* = 8.8275 (3) Å	µ = 0.08 mm^−^^1^
*c* = 18.7710 (7) Å	*T* = 120 K
β = 102.346 (1)°	Prism fragment, colorless
*V* = 1715.36 (10) Å^3^	0.72 × 0.61 × 0.44 mm
*Z* = 4	

### Data collection

**Table:**

Bruker APEXII CCD area-detector diffractometer	5009 independent reflections
Radiation source: fine-focus sealed tube	4876 reflections with *I* > 2σ(*I*)
graphite	*R*_int_ = 0.020
φ and ω scans	θ_max_ = 30.0°, θ_min_ = 2.0°
Absorption correction: multi-scan (*APEX2*; Bruker, 2006)	*h* = −14→14
*T*_min_ = 0.95, *T*_max_ = 0.96	*k* = 0→12
41010 measured reflections	*l* = 0→26

### Refinement

**Table:**

Refinement on *F*^2^	0 restraints
Least-squares matrix: full	H-atom parameters constrained
*R*[*F*^2^ > 2σ(*F*^2^)] = 0.042	*w* = 1/[σ^2^(*F*^2^) + ( 0.06*P*)^2^ + 0.63*P*] where *P* = (max(*F*_o_^2^,0) + 2*F*_c_^2^)/3
*wR*(*F*^2^) = 0.107	(Δ/σ)_max_ = 0.001
*S* = 1.00	Δρ_max_ = 0.37 e Å^−^^3^
4976 reflections	Δρ_min_ = −0.21 e Å^−^^3^
226 parameters	

### Fractional atomic coordinates and isotropic or equivalent isotropic displacement parameters (Å^2^)

**Table:**

	*x*	*y*	*z*	*U*_iso_*/*U*_eq_	
C1	0.94300 (8)	0.03374 (10)	0.99406 (5)	0.0188	
C2	0.86998 (8)	0.07821 (10)	0.91958 (5)	0.0172	
O3	0.91578 (6)	0.04194 (8)	0.86516 (3)	0.0213	
O4	0.76557 (6)	0.14853 (8)	0.91690 (4)	0.0229	
C5	0.63464 (8)	0.08298 (9)	0.73154 (4)	0.0158	
C6	0.53733 (8)	0.06749 (10)	0.66218 (4)	0.0155	
C7	0.53685 (9)	−0.06272 (10)	0.62033 (5)	0.0202	
C8	0.43586 (9)	−0.08628 (12)	0.56046 (5)	0.0247	
C9	0.33852 (9)	0.02100 (12)	0.54165 (5)	0.0239	
C10	0.34143 (8)	0.15261 (11)	0.58227 (5)	0.0220	
C11	0.44042 (8)	0.17580 (10)	0.64315 (5)	0.0189	
N12	0.59750 (7)	0.09379 (9)	0.79444 (4)	0.0177	
C13	0.47079 (8)	0.07070 (10)	0.80659 (4)	0.0171	
C14	0.43162 (9)	0.16661 (11)	0.85652 (5)	0.0223	
C15	0.30805 (10)	0.15224 (13)	0.87010 (5)	0.0268	
C16	0.22460 (9)	0.04177 (14)	0.83488 (5)	0.0282	
C17	0.26682 (10)	−0.05734 (13)	0.78741 (6)	0.0285	
C18	0.39004 (9)	−0.04455 (11)	0.77341 (5)	0.0230	
N19	0.76104 (7)	0.08174 (9)	0.73401 (4)	0.0182	
C20	0.82262 (8)	0.11807 (10)	0.67587 (4)	0.0173	
C21	0.77945 (8)	0.23541 (10)	0.62732 (5)	0.0200	
C22	0.84978 (9)	0.27416 (11)	0.57528 (5)	0.0232	
C23	0.96279 (10)	0.19753 (12)	0.57190 (5)	0.0259	
C24	1.00596 (10)	0.08164 (13)	0.62091 (6)	0.0305	
C25	0.93605 (9)	0.04137 (12)	0.67293 (6)	0.0260	
H11	0.9043	0.0548	1.0339	0.0224*	
H91	0.2695	0.0056	0.4998	0.0277*	
H101	0.2769	0.2275	0.5692	0.0264*	
H111	0.4430	0.2649	0.6712	0.0230*	
H141	0.4887	0.2423	0.8804	0.0269*	
H151	0.2816	0.2217	0.9032	0.0324*	
H161	0.1385	0.0352	0.8434	0.0313*	
H171	0.2125	−0.1372	0.7639	0.0336*	
H211	0.7042	0.2873	0.6302	0.0239*	
H221	0.8201	0.3536	0.5423	0.0286*	
H231	1.0108	0.2256	0.5354	0.0309*	
H241	1.0854	0.0287	0.6200	0.0370*	
H251	0.9653	−0.0391	0.7075	0.0301*	
H3	0.6030	−0.1359	0.6324	0.0240*	
H4	0.8118	0.0660	0.7773	0.0225*	
H8	0.4178	−0.1146	0.7427	0.0269*	
H9	0.6581	0.1188	0.8341	0.0217*	
H12	0.4343	−0.1762	0.5325	0.0293*	

### Atomic displacement parameters (Å^2^)

**Table:**

	*U*^11^	*U*^22^	*U*^33^	*U*^12^	*U*^13^	*U*^23^
C1	0.0190 (4)	0.0215 (4)	0.0147 (3)	−0.0001 (3)	0.0011 (3)	0.0002 (3)
C2	0.0163 (4)	0.0173 (4)	0.0164 (4)	0.0003 (3)	−0.0002 (3)	−0.0025 (3)
O3	0.0175 (3)	0.0294 (3)	0.0162 (3)	0.0017 (2)	0.0017 (2)	0.0008 (2)
O4	0.0186 (3)	0.0281 (3)	0.0196 (3)	−0.0029 (2)	−0.0010 (2)	0.0042 (2)
C5	0.0146 (3)	0.0169 (4)	0.0154 (3)	0.0006 (3)	0.0024 (3)	0.0014 (3)
C6	0.0138 (3)	0.0185 (4)	0.0141 (3)	0.0008 (3)	0.0026 (3)	0.0001 (3)
C7	0.0201 (4)	0.0209 (4)	0.0195 (4)	−0.0020 (3)	0.0037 (3)	0.0023 (3)
C8	0.0252 (4)	0.0269 (5)	0.0212 (4)	−0.0069 (3)	0.0035 (3)	−0.0027 (4)
C9	0.0197 (4)	0.0322 (5)	0.0180 (4)	0.0005 (3)	−0.0001 (3)	−0.0046 (3)
C10	0.0156 (4)	0.0261 (4)	0.0225 (4)	0.0048 (3)	0.0002 (3)	0.0010 (3)
C11	0.0166 (4)	0.0193 (4)	0.0200 (4)	0.0000 (3)	0.0021 (3)	0.0018 (3)
N12	0.0132 (3)	0.0248 (4)	0.0148 (3)	−0.0010 (3)	0.0024 (2)	0.0001 (3)
C13	0.0141 (3)	0.0228 (4)	0.0147 (3)	0.0021 (3)	0.0035 (3)	0.0011 (3)
C14	0.0222 (4)	0.0268 (4)	0.0192 (4)	−0.0026 (3)	0.0070 (3)	−0.0005 (3)
C15	0.0256 (5)	0.0334 (5)	0.0245 (4)	−0.0005 (4)	0.0125 (4)	0.0035 (4)
C16	0.0187 (4)	0.0434 (6)	0.0246 (4)	0.0046 (4)	0.0090 (3)	−0.0010 (4)
C17	0.0233 (4)	0.0391 (6)	0.0246 (4)	−0.0023 (4)	0.0083 (4)	−0.0108 (4)
C18	0.0223 (4)	0.0271 (4)	0.0212 (4)	−0.0030 (3)	0.0085 (3)	−0.0048 (3)
N19	0.0134 (3)	0.0260 (4)	0.0149 (3)	0.0024 (3)	0.0026 (2)	0.0023 (3)
C20	0.0147 (3)	0.0212 (4)	0.0161 (4)	−0.0003 (3)	0.0036 (3)	0.0000 (3)
C21	0.0189 (4)	0.0208 (4)	0.0207 (4)	0.0010 (3)	0.0049 (3)	0.0026 (3)
C22	0.0249 (4)	0.0241 (4)	0.0210 (4)	0.0036 (3)	0.0058 (3)	−0.0007 (3)
C23	0.0245 (4)	0.0315 (5)	0.0244 (4)	0.0019 (4)	0.0111 (3)	−0.0022 (4)
C24	0.0221 (4)	0.0375 (6)	0.0357 (5)	0.0076 (4)	0.0148 (4)	0.0074 (4)
C25	0.0196 (4)	0.0315 (5)	0.0288 (5)	0.0092 (4)	0.0093 (3)	0.0077 (4)

### Geometric parameters (Å, °)

**Table:**

C1—C1^i^	1.3223 (17)	C14—C15	1.3924 (13)
C1—C2	1.4982 (11)	C14—H141	0.947
C1—H11	0.944	C15—C16	1.3857 (15)
C2—O3	1.2625 (11)	C15—H151	0.957
C2—O4	1.2603 (11)	C16—C17	1.3892 (15)
C5—C6	1.4842 (11)	C16—H161	0.961
C5—N12	1.3255 (10)	C17—C18	1.3906 (13)
C5—N19	1.3304 (10)	C17—H171	0.956
C6—C7	1.3917 (12)	C18—H8	0.935
C6—C11	1.3926 (12)	N19—C20	1.4228 (11)
C7—C8	1.3919 (13)	N19—H4	0.884
C7—H3	0.944	C20—C21	1.3908 (12)
C8—C9	1.3892 (14)	C20—C25	1.3909 (12)
C8—H12	0.950	C21—C22	1.3922 (12)
C9—C10	1.3864 (14)	C21—H211	0.931
C9—H91	0.962	C22—C23	1.3886 (14)
C10—C11	1.3912 (12)	C22—H221	0.943
C10—H101	0.945	C23—C24	1.3864 (15)
C11—H111	0.944	C23—H231	0.969
N12—C13	1.4239 (10)	C24—C25	1.3922 (13)
N12—H9	0.901	C24—H241	0.966
C13—C14	1.3907 (12)	C25—H251	0.967
C13—C18	1.3882 (12)		
			
C1^i^—C1—C2	123.12 (10)	C15—C14—H141	120.5
C1^i^—C1—H11	119.4	C14—C15—C16	120.40 (9)
C2—C1—H11	117.5	C14—C15—H151	118.6
C1—C2—O3	118.51 (8)	C16—C15—H151	121.0
C1—C2—O4	116.12 (8)	C15—C16—C17	119.28 (9)
O3—C2—O4	125.36 (8)	C15—C16—H161	119.7
C6—C5—N12	120.33 (7)	C17—C16—H161	121.0
C6—C5—N19	122.37 (7)	C16—C17—C18	120.95 (9)
N12—C5—N19	117.26 (7)	C16—C17—H171	121.1
C5—C6—C7	119.29 (7)	C18—C17—H171	118.0
C5—C6—C11	119.85 (8)	C17—C18—C13	119.23 (9)
C7—C6—C11	120.58 (8)	C17—C18—H8	119.7
C6—C7—C8	119.25 (8)	C13—C18—H8	121.1
C6—C7—H3	121.1	C5—N19—C20	126.50 (7)
C8—C7—H3	119.7	C5—N19—H4	116.3
C7—C8—C9	120.28 (9)	C20—N19—H4	116.8
C7—C8—H12	119.4	N19—C20—C21	122.00 (8)
C9—C8—H12	120.4	N19—C20—C25	117.56 (8)
C8—C9—C10	120.23 (8)	C21—C20—C25	120.17 (8)
C8—C9—H91	120.3	C20—C21—C22	119.49 (8)
C10—C9—H91	119.4	C20—C21—H211	119.6
C9—C10—C11	119.95 (8)	C22—C21—H211	120.9
C9—C10—H101	120.9	C21—C22—C23	120.57 (9)
C11—C10—H101	119.1	C21—C22—H221	119.3
C6—C11—C10	119.66 (8)	C23—C22—H221	120.1
C6—C11—H111	119.9	C22—C23—C24	119.65 (9)
C10—C11—H111	120.5	C22—C23—H231	119.7
C5—N12—C13	127.02 (7)	C24—C23—H231	120.6
C5—N12—H9	117.3	C23—C24—C25	120.28 (9)
C13—N12—H9	115.7	C23—C24—H241	120.7
N12—C13—C14	116.64 (8)	C25—C24—H241	119.1
N12—C13—C18	123.03 (8)	C24—C25—C20	119.83 (9)
C14—C13—C18	120.29 (8)	C24—C25—H251	121.0
C13—C14—C15	119.73 (9)	C20—C25—H251	119.2
C13—C14—H141	119.8		

Symmetry codes: (i) −*x*+2, −*y*, −*z*+2.

### Hydrogen-bond geometry (Å, °)

**Table:**

*D*—H···*A*	*D*—H	H···*A*	*D*···*A*	*D*—H···*A*
N19—H4···O3	0.88	1.79	2.673 (1)	178
N12—H9···O4	0.90	1.74	2.634 (2)	172
